# Imaging Poliovirus Entry in Live Cells

**DOI:** 10.1371/journal.pbio.0050183

**Published:** 2007-07-10

**Authors:** Boerries Brandenburg, Lily Y Lee, Melike Lakadamyali, Michael J Rust, Xiaowei Zhuang, James M Hogle

**Affiliations:** 1 Department of Chemistry and Chemical Biology, Harvard University, Cambridge, Massachusetts, United States of America; 2 Howard Hughes Medical Institute, Cambridge, Massachusetts, United States of America; 3 Department of Biological Chemistry and Molecular Pharmacology, Harvard Medical School, Boston, Massachusetts, United States of America; 4 Department of Physics, Harvard University, Cambridge, Massachusetts, United States of America; University of Wisconsin-Madison, United States of America

## Abstract

Viruses initiate infection by transferring their genetic material across a cellular membrane and into the appropriate compartment of the cell. The mechanisms by which animal viruses, especially nonenveloped viruses, deliver their genomes are only poorly understood. This is due in part to technical difficulties involved in direct visualization of viral gene delivery and to uncertainties in distinguishing productive and nonproductive pathways caused by the high particle-to–plaque forming unit ratio of most animal viruses. Here, we combine an imaging assay that simultaneously tracks the viral capsid and genome in live cells with an infectivity-based assay for RNA release to characterize the early events in the poliovirus (PV) infection. Effects on RNA genome delivery from inhibitors of cell trafficking pathways were probed systematically by both methods. Surprisingly, we observe that genome release by PV is highly efficient and rapid, and thus does not limit the overall infectivity or the infection rate. The results define a pathway in which PV binds to receptors on the cell surface and enters the cell by a clathrin-, caveolin-, flotillin-, and microtubule-independent, but tyrosine kinase- and actin-dependent, endocytic mechanism. Immediately after the internalization of the virus particle, genome release takes place from vesicles or tightly sealed membrane invaginations located within 100–200 nm of the plasma membrane. These results settle a long-lasting debate of whether PV directly breaks the plasma membrane barrier or relies on endocytosis to deliver its genome into the cell. We expect this imaging assay to be broadly applicable to the investigation of entry mechanisms for nonenveloped viruses.

## Author Summary

During travel between hosts, the genome of a virus is well protected by the viral capsid and/or envelope. After binding specifically to target cells, the virus particles enter cells by hijacking cell trafficking pathways and then deliver the viral genome into the appropriate compartment of the cell where it directs the production of progeny virus particles. How nonenveloped viruses, such as poliovirus, enter target cells is not well understood. Here, we produced fully infectious poliovirus with both genome and capsid specifically labeled by fluorescent dyes. We could then use real-time fluorescent microscopy to follow single virus particles during infection, to define how they enter cells and to determine when and where in the cell the genome gets released. We have complemented the microscopic studies with virological assays, which demonstrate that the pathways observed by microscopy are productive. We show that poliovirus enters live cells in a process that requires energy, an intact actin cytoskeleton, and cell signaling pathways, but does not depend on the well-known markers of endocytic pathways. We show that after internalization, the genome release is surprisingly efficient and occurs from vesicles that are very close to the cell surface. Our experiments offer new insights into the early steps of poliovirus infection, and describe methods that can be used for a wide variety of other viruses.

## Abstract 3

Combining an imaging assay that simultaneously tracks the viral capsid and genome in live cells with an infectivity-based biological assay for RNA release, the authors settle a long-lasting debate on the nature of poliovirus entry into the host cell.

## Introduction

As obligatory intracellular parasites with limited genetic capacity, viruses have evolved to hijack intrinsic cellular pathways to enter the cell and deliver their genomes to specific cellular locations for replication. Therefore, mechanistic understandings of viral entry may not only lead to new therapies for combating viral infection, but also provide new insights into fundamental cellular functions [1]. A number of distinct strategies have been exploited for viral entry and gene delivery. For enveloped viruses, protein-assisted fusion of viral and cellular membranes provides a conceptually simple mechanism for capsid or genome release into the cytoplasm [2]. For nonenveloped viruses, the mechanism is less well understood, but appears to rely upon viral capsid proteins (VPs) to disrupt cellular membranes or to form pores through them [3]. The cellular sites where genome release occurs are unknown for most nonenveloped viruses.

Here, we chose poliovirus (PV) as a model system to study entry and genome delivery by nonenveloped viruses. PV is a picornavirus that causes human poliomyelitis and is closely related to other important human viral pathogens, including rhinoviruses, coxsackieviruses, echoviruses, and enteroviruses. The virion is comprised of an icosahedral capsid, harboring a positive-sensed single-stranded RNA (~7.5 kilobases) [4].

PV infection is initiated when the virus binds the poliovirus receptor (PVR, or CD155) [5]. At physiological temperature, the binding of multiple PVRs triggers an irreversible conformational change in the native virion (160S particle), resulting in the formation of an altered particle (135S) [6]. This conformational change results in externalization of myristoylated capsid protein VP4 [6,7] and the N-terminus of the capsid protein VP1 [8]. Both of the externalized peptides then insert into membranes [8,9], allowing the virus particle to anchor to the cellular membrane in a receptor-independent manner [8,10] and to form channels and pores in planar membranes [9,11,12]. This has led to the suggestion that the membrane-associated viral peptides facilitate translocation of the viral genome across the plasma or vesicle membrane and into the cytoplasm. Genome release results in the formation of a stable empty particle (the 80S particle) [13].

The pathway by which PV enters cells is unclear. Early studies using electron microscopy, cell fractionation, lysotrophic amines, and inhibitors of endocytosis suggested that PV enters cells via clathrin-mediated endocytosis and that viral uncoating depends on acidification of early endosomes [14–18]. In contrast, more-recent studies have demonstrated that PV infection is not affected by expression of dominant negative mutants of the protein dynamin (which is required for maturation of clathrin-coated vesicles and caveolae) [19] or by treatment with bafalomycin A1 (a specific inhibitor of the vacuolar proton pump); but that entry is blocked by co-administration of valinomycin, an ionophore that induces potassium ion efflux, and concanamycin A, a vacuolar proton ATPase inhibitor that prevents endosomal acidification [20]. The conflicting results highlight several fundamental difficulties associated with characterizing viral entry pathways: (1) the high particle-to–plaque forming unit (pfu) ratio of animal viruses (100:1 to 200:1 for PVs, and higher for many other viruses) makes it difficult to distinguish productive from nonproductive pathways, (2) conventional biochemical and visualization assays generally require a very high multiplicity of infection (MOI), increasing the probability of sampling nonproductive pathways, and (3) inhibitors of cell trafficking pathways (including small molecules, small interfering RNAs [siRNAs], and dominant negative mutants) may have poorly characterized secondary targets that affect events downstream of the entry process. Consequently, despite decades of study, the PV cell entry pathway remains a mystery. Indeed, even questions such as whether the virus directly releases its genome across the plasma membrane or hijacks an endocytic pathway to deliver the genome from an intracellular organelle or vesicle remain unanswered.

In this work, we characterized the PV entry pathway by combining live-cell imaging of infectious dual-labeled PV in which the viral capsid and RNA were labeled with distinct fluorophores with biochemical assays that directly measure RNA release. These assays were used to characterize early events in infections initiated at a low MOI in the presence or absence of inhibitors of cell trafficking pathways. We found that PV RNA release was a rapid and efficient process that did not limit the specific infectivity of the virus. After binding to the cell, the virus particles were internalized in an actin-dependent manner. The genome release took place only after endocytosis, in vesicles very close (within 100–200 nm) to the plasma membrane. The kinetics of RNA release in the visualization assay and the biological assay were indistinguishable, and both assays demonstrated that RNA release was energy-, actin-, and tyrosine kinase-dependent, but did not require clathrin, caveolin, flotillin, or microtubules.

## Results

### Capsid and Genome Dual-Labeled PV

Simultaneous visualization of viral capsid and genome requires the identification of distinct fluorescent labels for the capsid proteins and genome RNA that do not reduce the specific infectivity of the virus. The capsid of purified PV can be readily labeled with amine-reactive dyes (e.g., Cy5, Cy3, and Alexa Fluor 633). Unfortunately, the RNA genome, once incorporated into the virion, is not accessible to dye attachment even when the dyes and virions are co-incubated at temperatures permissive for capsid “breathing,” a phenomenon that has previously been shown to allow incorporation of dyes such as Ribogreen into rhinoviruses [21,22]. We therefore turned to a metabolic labeling strategy to incorporate dye-labeled RNA during the viral replication process. A similar approach has been used to introduce neutral red (NR) and acridine orange into virions [23,24], but these dyes are unsuitable for imaging because they rapidly inactivate the viral RNA upon illumination. More than 20 RNA-binding dyes, including Ribogreen, RNA Select, and a series of Syto dyes from Molecular Probes, were screened by the following criteria: (1) the dye must be membrane permeable, (2) it must be noncytotoxic, (3) it must be able to bind viral RNA at sufficient levels to allow single RNA visualization, (4) it must not interfere with RNA incorporation into virions, and (5) it must not reduce the specific infectivity of the virus. Only one dye, Syto82 (excitation_max_ = 541 nm, and emission_max_ = 571 nm), met all five criteria. Experiments with in vitro–transcribed PV RNA showed that unfolding the RNA substantially reduced the fluorescent signal of Syto82 originally associated with the RNA (unpublished data), suggesting that RNA secondary structure is important for Syto82 binding, consistent with the property of intercalating dyes. We chose Cy5 (excitation_max_ = 649 nm, and emission_max_ = 670 nm) as the complementary capsid label.

**Figure 1:**
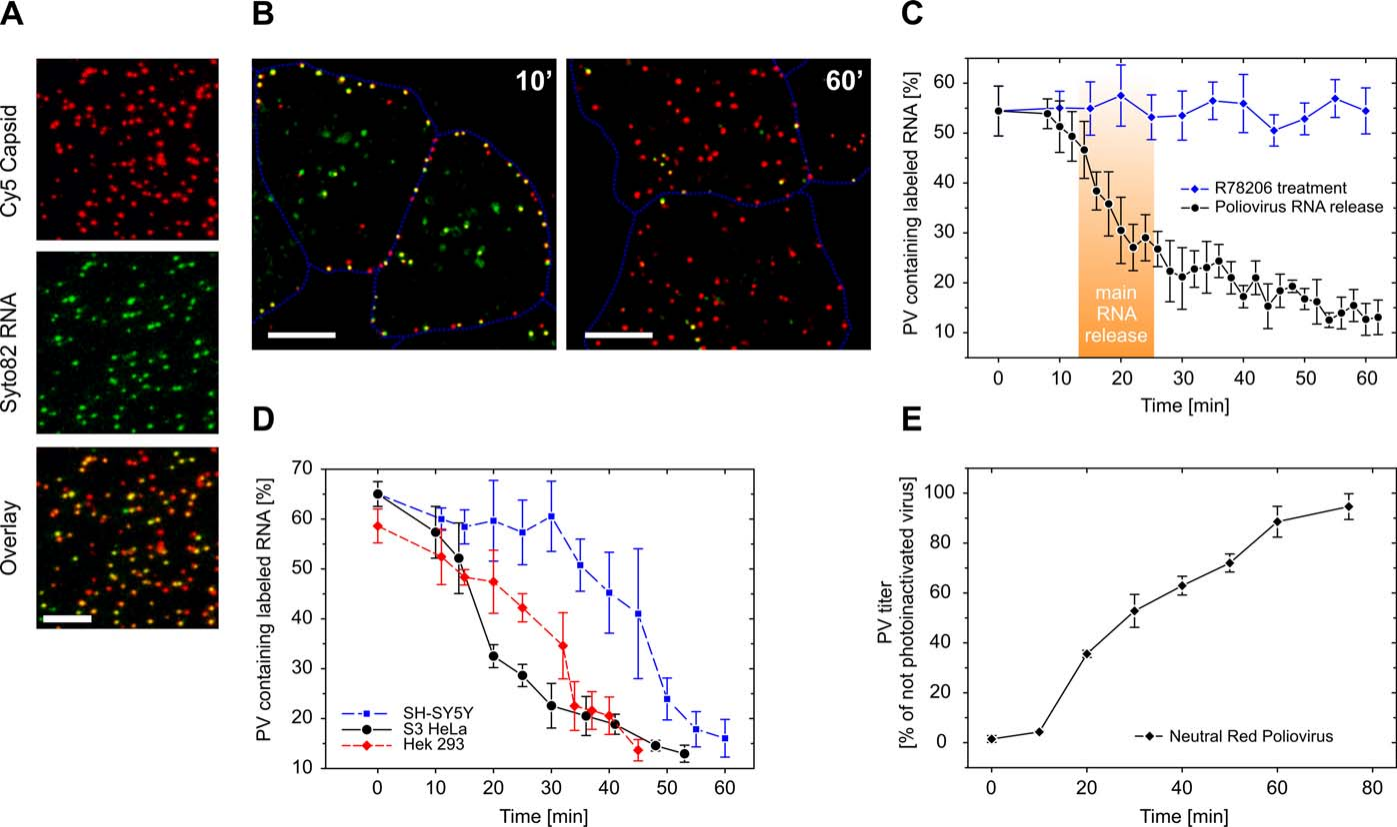
Rapid and Efficient RNA Release of PV (A) Dual-labeled PV genomic RNA, labeled with Syto 82 (green) colocalizes with the Cy5 labeling of the viral capsid (red). Scale bar indicates 5 μm. (B) Imaging of RNA release in live HeLa cells at the single virus particle level. Cells infected with dual-labeled PV and imaged 10 min and 60 min p.i.. Scale bar indicates 10 μm. (C) PV RNA release kinetics in HeLa cells. The cells were infected with PV at MOI = 1. The fraction of Cy5-positive particles containing Syto82 signal was detected and counted at different time points postinfection. In contrast to the R78206 negative control (R78206 specifically binds PV capsid and inhibits conformational change and RNA release), untreated dual-labeled PV releases RNA efficiently. (D) RNA release could be observed in several cell types with only moderate kinetics delays. (E) RNA release kinetics measured in the NR assay. At various time points, the cells infected with NR-labeled viruses were exposed to white light, inducing NR crosslinking of the viral RNA only while the dye and RNA are constrained within the virus particle. Upon RNA release, the dye and RNA are no longer constrained, and the infection becomes insensitive to irradiation. The plot shows the titer numbers measured in cells exposed to light at various time points postinfection.

The protocols for labeling the capsid and RNA were optimized such that both dyes were incorporated to yield the highest level of fluorescent signal without appreciably affecting PV infectivity (Figures 1A and S1). Under these conditions, the average number of Cy5 dye molecules per PV particle was estimated to be 40–50. We thus expect nearly 100% of the virus particles to be Cy5 positive. Consistent with this hypothesis, nearly 100% of the virions with Syto82 signal also exhibited Cy5 signal. However, 55%–65% of the Cy5-positive virions showed Syto82 signal. The remaining virions presumably did not contain a sufficient amount of Syto82 for single-particle detection.

### Genome RNA Release of PV Is Rapid and Efficient

The highly efficient labeling of viral capsid and RNA allowed us to monitor the RNA release in live cells at the single-virion level. To this end, we infected human cervical carcinoma-derived cells (HeLa S3) with the dual-labeled virus at a low MOI (1 pfu/cell), which allows us to discern individual virus particles. About 300–400 PV particles were observed bound per cell. After incubation with cells at 37 °C, the bound virus particles lose their RNA signal over time (Figure 1B). A time course of this process can be directly visualized from images of the same cell taken at different time points (Figure S2A and S2B). The same experiment conducted at lower temperature (24 °C) showed little RNA signal loss (Figure S2C and S2D), suggesting that the fluorescence loss was not due to photobleaching of the RNA label, but rather due to RNA release. In this experiment, short exposure-time images (500 ms) were recorded only every few minutes to avoid extensive photobleaching of the Syto82 dye and photodamage of the dye-labeled RNA. In order to ensure that any potential effect of photodamage was excluded, we also adopted an alternative imaging procedure and changed the imaging area after each time point, such that no cells were imaged more than once or ever exposed to excitation prior to imaging. A similar RNA release kinetics curve was obtained (Figure 1C). The viruses began to lose their RNA signal after an initial lag of about 10 min, and the fraction of Cy5-positive particles containing Syto82 signal showed a steep descent between 10 and 30 min. As a negative control, we used the Janssen drug R78206 [25], which binds specifically to PV capsids and prevents the receptor-induced conformational change and subsequent RNA release. In the presence of R78206, the fraction of Cy5-positive particles containing Syto82 signal remained constant throughout the experiment (Figure 1C), suggesting that the loss of RNA label is indeed due to the release of RNA from the virus particles. Interestingly, the loss of Syto82 signal from the Cy5-positive capsids was not accompanied by the appearance of single-colored Syto82 spots elsewhere, whereas the binding of Syto82 to in vitro–transcribed PV RNA at physiological salt concentrations was found to be stable for several hours. We thus propose that RNA release requires disruption of the RNA secondary structure leading to the release of the RNA dye upon externalization.

Surprisingly, RNA release was highly efficient. At the outset of infection, roughly 60% of the Cy5-labeled virus contained the RNA label. By 60 min postinfection (p.i.), the percentage was reduced to 10%–15% (Figure 1C). RNA release in HeLa cells occurred with a *t*_1/2_ of 22 ± 3 min. We also analyzed the RNA release kinetics in human kidney-derived cells (Hek 293) and human neuroblastoma cells (SH-SY5Y). Although the kinetics of release was slower in Hek 293 and in SH-SY5Y cells, RNA release was efficient in all three cell types (Figure 1D).

To further verify the results of the imaging assay, we performed an infection-based assay for RNA release using NR-containing virus. NR is readily incorporated into virions grown in cells containing the dye. It has been shown that exposure to light can cause NR dye to crosslink viral RNA when both are contained within the capsid, rendering the virus particle noninfectious (Figure S1) [23,24]. Upon RNA release, the dye is detached from the RNA and the infection becomes insensitive to irradiation. This assay has been used to probe the effects of mutations and drugs on the kinetics of RNA release [16,26]. The kinetics of RNA release as measured by this assay (*t*_1/2_ between of 27 ± 3 min) closely mirrored the kinetics of RNA release as measured by the visualization assay (Figure 1E, see also [9]), supporting that the RNA release visualized by our imaging assay is relevant to infection.

### Genome Release Occurs after Virus Internalization from Vesicles near the Plasma Membrane

**Figure 2:**
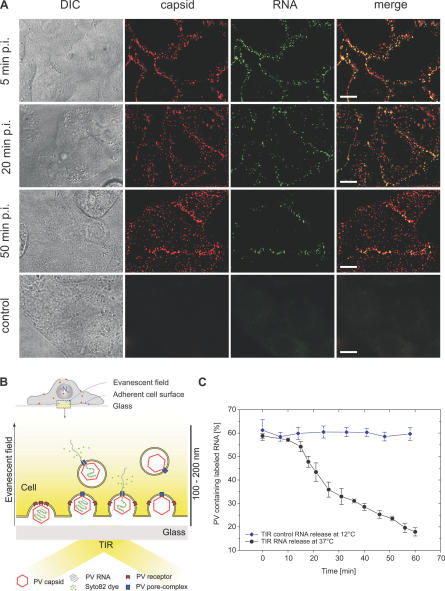
PV RNA Release Occurs near the Cell Surface (A) Live HeLa cells at different times postinfection with dual-labeled PV (MOI = 2–3). During the infection, the number of capsids (red) observed inside the cell is increasing. Colocalization with the RNA (green) is observed primarily near the cell surface and rarely seen deep inside the cell. Scale bar indicates 10 μm. DIC, differential interference contrast. (B) By using TIRF microscopy, it is possible to investigate RNA release kinetics within the 100–200 nm of the evanescent field at the adherent surface of the cell. (C) PV RNA releases observed under TIRF imaging condition.

Confocal images of cells infected with dual-labeled virus at various times p.i. provided us with the first glimpse of the PV RNA release sites. At an early point (5 min p.i.) before any substantial RNA release took place, nearly all of the labeled viruses remained close to the cell surface. Only at later time points (20–50 min p.i.) was there an accumulation of virus particles in the inner part of the cell body. These particles were nearly exclusively singly labeled, containing only the capsid (Cy5), but not the RNA (Syto82) label, whereas a substantial fraction of the particles near the cell surface remained both capsid and RNA positive (Figure 2A). These images were taken at a higher MOI (2–3 pfu/cell) for visual demonstration, but were not used for single-virus counting analysis. Similar results were observed at a lower MOI at which individual virus particles are discernable, suggesting that RNA release primarily took place near the cell surface; and afterwards, empty capsids were transported towards more-interior regions of the cell.

Immediately after inoculation, PV particles were first observed at the top surface of the cell and began to appear at the bottom adhering surface only after a short delay of 1–3 min. Previous studies on polarized cells have shown that PV is able to enter via both the apical and the basolateral surface [27]. We therefore analyzed the *z*-scans to separately assess the rates of RNA release from the top and bottom surfaces of live cells. Both surfaces exhibited similar kinetics of RNA release (unpublished data). This allowed us to image genome release from the adherent cell surface using total internal reflection fluorescence (TIRF) microscopy (Figure 2B). The evanescent wave produced by total internal reflection generates an illumination field of only 100–200 nm deep from the adhering cell surface, allowing us to precisely determine the proximity of RNA release sites to the plasma membrane. In these experiments, a low MOI (1 pfu/cell) was used to allow virus particle counting. We found that RNA was efficiently released within the TIR illumination field in both experiments in which the same cell was repetitively imaged (Figure S2C) or in which the imaging area was changed after each time point (Figure 2C), indicating that genome release was taking place within 100–200 nm of the cell surface. As a control, we conducted the same imaging assay at 12 °C, a temperature at which infection does not occur [28]. The fraction of particles containing the RNA label remained constant throughout the experiment (Figure 2C), indicating the genome release is temperature dependent.

**Figure 3:**
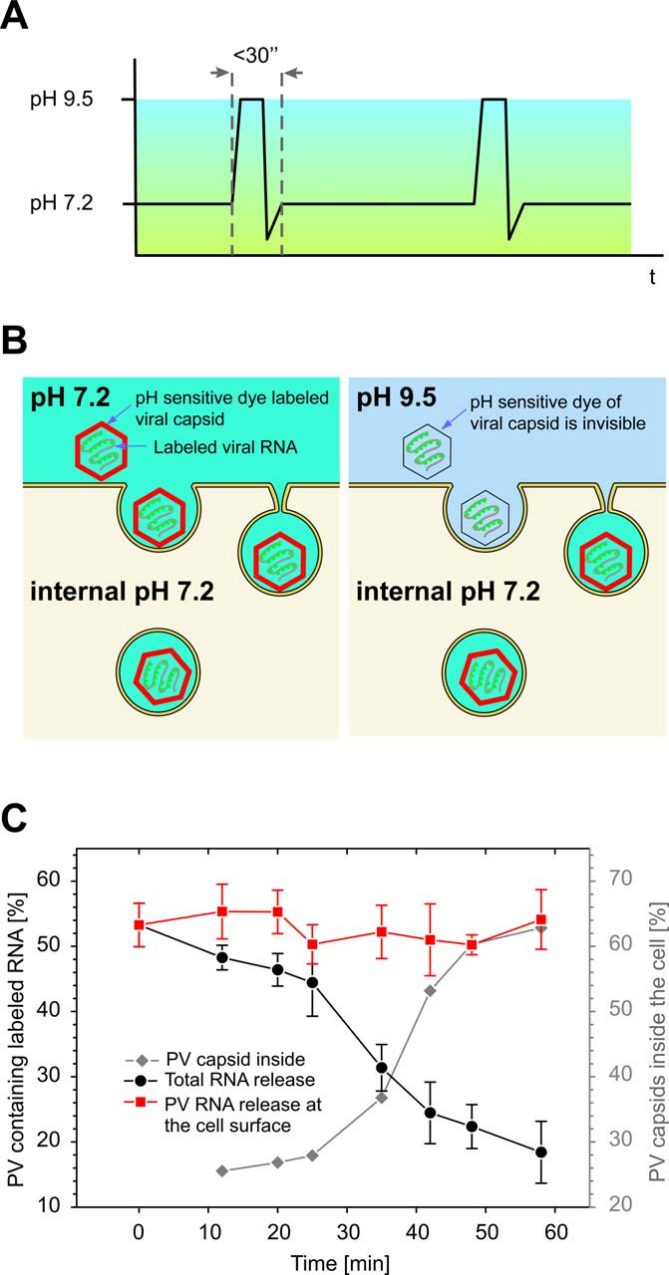
PV RNA Release Occurs after Virus Internalization The pH-sensitive CypHer5 PV capsid labeling allows distinction between the population of viruses outside versus inside the cell. (A) Live cells were pulsed during the infection with pH 9.5 medium. (B) CypHer5 becomes nonfluorescent at pH 9.5, resulting in the disappearance of capsid signals outside the cell (black capsid) during the pulse. Viruses that have already entered the cell are protected by the buffering capacity of living cells and are still observable (red capsid). (C) Tracking pH-sensitive PV at different times postinfection showed that the RNA release kinetics (black) was similar to the rate of increase for the number of internalized capsids (grey). Only viruses that have entered the cell are able to release RNA. Capsids on the surface do not release their RNA (red).

In order to determine whether RNA release was taking place at the plasma membrane or from internalized vesicles close to the cell surface, we performed an experiment in which the capsid was labeled with a pH-sensitive dye, CypHer5, which is brightly fluorescent at neutral and acidic pH values (pH ≤ 7) but becomes essentially nonfluorescent at pH ≥ 9. Cells infected with dual-labeled virus were pulsed with pH 9.5 media at various time points (Figure 3A). Because a living cell is able to buffer its internal pH, only the population of PV outside the cell would be sensitive to changes in the extracellular pH (Figure 3B). The kinetics of RNA release was similar to those observed previously with Cy5-labeled viruses in neutral media (Figure 3C). The percentage of input virus particles sensitive to external pH change decreased as a function of time, indicating the internalization of the virus particles. The measured internalization kinetics closely mirrored the RNA release kinetics (Figure 3C). These results indicate that the RNA release occurred roughly at the same time the virus particles were internalized. Remarkably, RNA release was not observed among the virus particles that remained on the cell surface and sensitive to external pH change (Figure 3C, red curve), indicating that RNA release only occurred after internalization or at least after the virion become sheltered from the extracellular medium. Taken together, these results suggest that PV must first enter the cell before releasing its genome and that the genome release occurs quickly afterward, within a distance of 100–200 nm of the plasma membrane.

### An Infectivity-Based Assay for RNA Release

**Figure 4:**
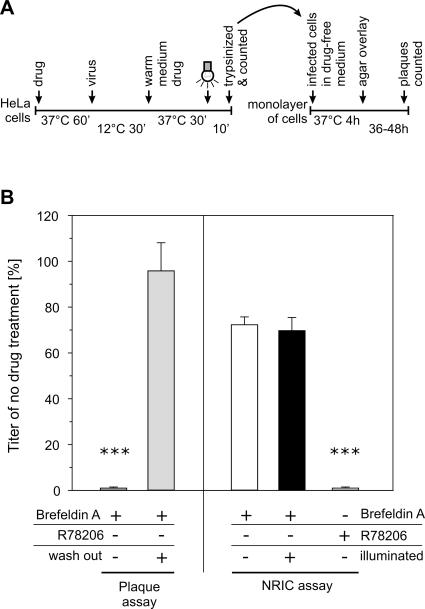
The Neutral Red Infectious Center Assay (A) Schematic for the NRIC assay. Cells were pretreated with an inhibitor for 1 h prior to infection in the dark with NR PV. Infection was initiated by warming to 37 °C. In order to probe for inhibitor effects only during the early steps of infection, the cells were exposed to white light at 30 min p.i. to inactivate intact viruses, the inhibitor was removed, and the cells were trypsinized. The detached cells were used to infect a fresh monolayer of HeLa cells in the absence of inhibitor, and the plaques were counted at 2 d p.i.. (B) Brefeldin A is known to inhibit later stages of PV infection and was used to demonstrate the ability of the NRIC assay to exclusively show inhibitor effects during the early stage of PV infection. In contrast to a normal plaque assay, Brefeldin A in the NRIC assay has no effect on the infection. R78206 was used as a positive control that irreversible binds and inactivates the PV capsid. Triple asterisks (***) indicate *p* < 0.001.

Whereas imaging labeled viruses in living cells provides a powerful assay to probe the kinetics and sites of viral genome release, this type of assay also has a significant limitation—it does not directly show whether the observed RNA release leads to infection. In order to establish the biological significance of the results obtained using the visualization assay, we refined a neutral red–dependent infectious center (NRIC) assay [16] to specifically target early infection steps leading to the viral RNA release. In the refined NRIC assay (Figure 4A), HeLa cells were pretreated with a given inhibitor for 1 h prior to infection with NR-labeled PV at an MOI of one. At 30 min p.i., the cells were exposed to light to inactivate viruses that had not yet released RNA. The drugs were then removed, and the inoculated cells were then seeded onto a fresh layer of cells in the absence of inhibitor, and the percentage of cells infected was determined by plaque assay. In a parallel assay, the light illumination step was omitted to control for irreversible effects of the inhibitor on the cell or the virus.

In order to confirm that the assay is able to distinguish between the effect of drugs on RNA release and late stages of infection, we used the assay to assess the effect of two drugs: the capsid-binding drug R78206, a drug known to block conformational changes and RNA release (Figure 1C) [25]; and brefeldin A, a drug that disrupts endoplasmic reticulum to Golgi trafficking that has been shown to block PV RNA replication late in infection, but has no effect on early events [29]. As expected, the PV-specific inhibitor R78206 caused severe and irreversible reduction in titer in the NRIC assay (Figure 4B). Consistent with earlier findings, brefeldin A had a pronounced effect on viral replication in a conventional plaque assay when present throughout infection, but had no effect on infection in the NRIC assay or in the plaque assay if the drug was washed out. We combined the imaging assay and NRIC assay to probe the effects of a panel of inhibitors of cell trafficking pathways to gain additional insights into the mechanism of PV entry, as discussed below.

### The PV Genome Release Is ATP Dependent

**Figure 5:**
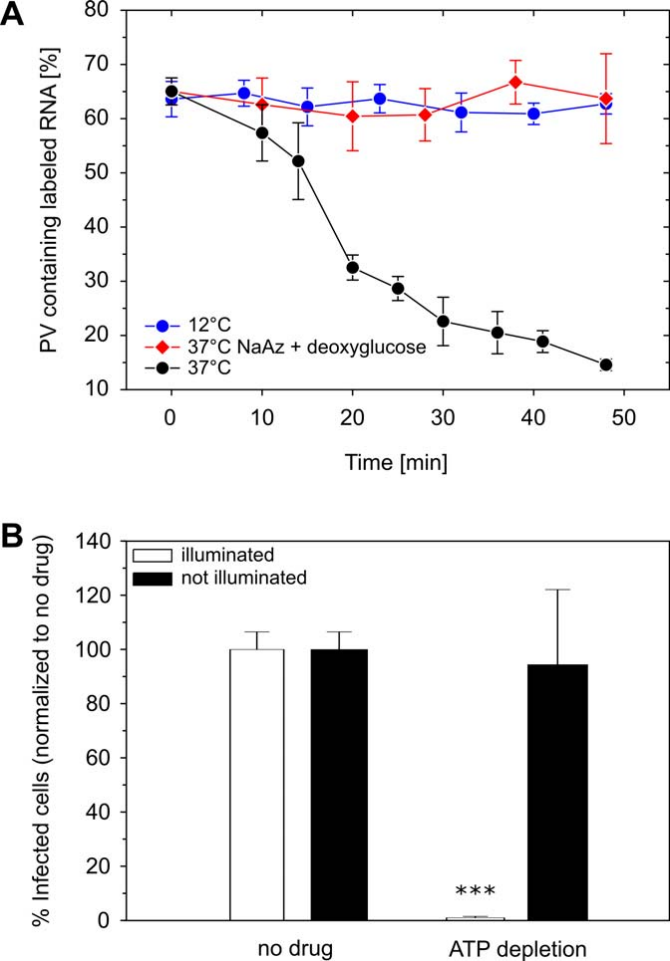
PV Entry Is Temperature and Energy Dependent (A) The single-virus imaging assay shows that PV RNA release does not occur at nonphysiological temperatures (12 °C) and depends on the presence of cellular ATP that can be depleted by treating the cells with sodium azide (NaAz) and deoxyglucose. (B) The ATP dependence of PV RNA release shown in the NRIC assay. Triple asterisks (***) indicate *p* < 0.001.

Endocytosis is an energy-dependent process. The observation that RNA release only occurs after internalization of the virus particles suggests that the release of PV RNA would be energy dependent. To test this hypothesis, we depleted cellular ATP by pretreating the cells with sodium azide and 2-deoxyglucose. Depletion was confirmed by the absence of mitochondrial staining with rhodamine 123 [30]. As expected, PV was unable to release its RNA in ATP-depleted cells (Figure 5A). Consistent with this result, the RNA release also did not proceed at 12 °C, a temperature at which endocytosis is not permitted. Using the NRIC assay, we observed a severe reduction in the percentage of cells infected when ATP was depleted using sodium azide and 2-deoxyglucose (Figure 5B), but the reduction was not observed when the illumination step was omitted. These observations demonstrate that the steps leading to productive RNA release are ATP dependent.

### PV Entry Depends on Actin, but Not Microtubules

**Figure 6:**
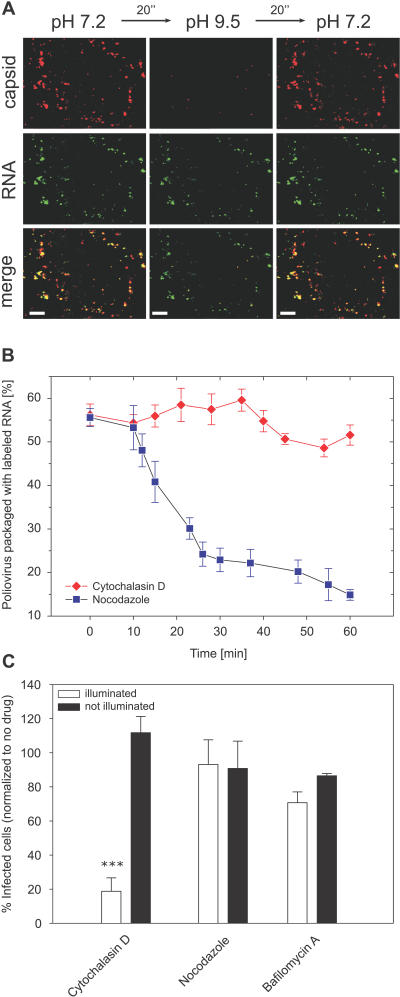
PV Entry Is Actin Dependent (A) Actin-cytoskeleton is required for PV internalization and RNA release. Cells were pretreated with an actin-disrupting drug, Cyto-D. By briefly changing the pH in the medium to pH 9.5, signals of nearly all of the pH-sensitive CypHer5-labeled viral capsids disappeared at 60 min p.i., indicating that the virus particles were still outside of the cells. The RNA label (Syto82, green) was also detectable in nearly all of the virus particles, indicating no RNA release in these particles. In contrast, in untreated cells, the majority of viruses have entered the cell and released their RNA genome by 60 min p.i. (Figure 3C). Scale bar indicates 10 μm. (B) The single-virus imaging assay shows that PV RNA release is inhibited by Cyto-D, but not by the microtubule-depolymerizing drug, nocodazole. (C) Similarly, RNA release is inhibited by Cyto-D, but not by nocodazole or bafalomycin A1 (which inhibit vacuolar proton pump and thus inhibit endosome acidification) in the NRIC assay. Triple asterisks (***) indicate *p* < 0.001.

Many endocytosis processes are actin dependent [31]. To further test the idea that PV genome release takes place after endocytosis of the virus particle, we sought to test the actin dependence of RNA release. To this end, we treated the cells with cytochalasin D (Cyto-D), a drug that causes actin depolymerization. As visualized in the live-cell imaging assay, virus particles still bound in similar quantities to the treated cells as to the untreated cells, suggesting that Cyto-D treatment did not affect PV–PVR interactions. To test whether the internalization of the virus particles were inhibited by Cyto-D, we again exploited our CypHer 5–labeled PV (Figure 6A). In contrast to untreated cells in which the virus label rapidly became insensitive to extracellular pH change, the great majority of virus particles added to Cyto-D–treated cells remained sensitive to the pH change throughout the experiment (60 min), indicating that the internalization of PV is indeed inhibited by actin disruption.

Remarkably, the fraction of virus particles containing Syto82 remained constant throughout the entire course of the experiment (60 min), indicating that genome release was severely inhibited by disrupting actin cytoskeleton (Figure 6A and 6B). Similarly, we observed a significant decrease in the titers measured in Cyto-D–treated cells using the NRIC assay as compared to control cells (Figure 6C). These results indicate that productive RNA release is actin dependent, consistent with the notion that PV RNA release requires internationalization of the viral particle.

In contrast, RNA release was shown by both live-cell imaging and NRIC assay to be unaffected by nocodazole, which inhibits microtubule-dependent transport in cells (Figure 6B and 6C). The lack of microtubule dependence in both assays suggests that the productive RNA release is unlikely to be dependent on the acidic environment of late endosomes and lysosomes. Consistent with this conclusion, treatment with bafilomycin A1, a vacuolar proton ATPase inhibitor that prevents endosome acidification, had no effect on PV infectivity (Figure 6C). The lack of dependence of RNA release on microtubules and acidification of endosomes is consistent with previously published results [18,32,33]. After RNA release, empty capsids almost exclusively labeled with Cy5 only were found to rapidly move in a microtubule-dependent manner towards the cell center. This behavior should thus be irrelevant to infection, and may suggest that the empty capsids are transported to compartments with a degradative environment, such as late endosomes and lysosomes. Trafficking of endocytic ligands to these organelles is typically microtubule dependent [34].

### PV Infection Does Not Require Clathrin, Caveolin, Flotillin, or Macropinocytosis, but Depends on Tyrosine Kinases

**Figure 7:**
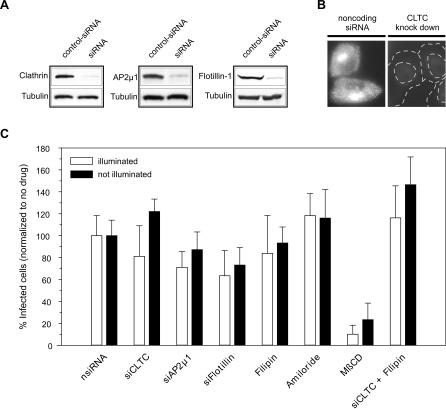
Endocytosis of PV Is Independent of Clathrin, Caveolin, Flotillin, and Macropinocytosis (A) The knockdown effect of siRNA against the clathrin heavy chain (siCLTC), the μ1 subunit of the adaptor protein 2 (siAP2μ1) and flotillin-1 (siFlotillin) is shown by Western blot analysis. (B) As a control, the uptake of Alexa Fluor 633–labeled transferrin was blocked in cells transfected with the CLTC siRNA (right) but not with noncoding siRNA (left). Fluorescent transferrin was incubated with the HeLa cells for 40 min. Transferrin molecules remaining on the cell surface were removed by an acid wash before imaging. (C) Effect of siRNAs and small-molecule inhibitors on PV entry measured by using the NRIC assay. nsiRNA, noncoding siRNA.

Having established that the RNA release occurs only after internalization, a natural question arises as to what endocytic mechanism is exploited by PV. A number of endocytic portals were used by animal viruses to gain entry into the cell, including clathrin-mediated endocytosis, caveolin-mediated endocytosis, clathrin- and caveolin-independent pathways, and macropinocytosis [1,35]. To characterize which pathway(s) may be used by PV, we used the NRIC assay to test the effect of previously established endocytic inhibitors (Figure 7).

Although chlorpromazine is commonly used to inhibit clathrin-mediated endocytosis, this drug also elicits other secondary effects on the cell, including inhibition of voltage-gated potassium channels in neuronal cells, and inhibition of lysosomal and cytosolic phospholipases [36,37]. In the NRIC assay, chlorpromazine treatment resulted in similar levels of reduction in infection in both the illuminated cells and nonilluminated controls, suggesting that these or other side effects of the drug dominate the decrease in titer (unpublished data). To specifically inhibit clathrin-mediated endocytosis, we used siRNA to knockdown the expression level of clathrin heavy chain (CLTC) or that of the μ1 subunit of AP2 complex. Both siRNAs have been previously used to inhibit clathrin-mediated endocytosis [38]. Knockdown was confirmed by Western blot analysis (Figure 7A). As a positive control, we confirmed that the uptake of fluorescently labeled transferrin was blocked in the CLTC siRNA-transfected cells, but not in cells transfected with noncoding siRNA (Figure 7B). In contrast to the marked effect on transferrin uptake, the knockdown of CHC or AP2 had no significant effect on PV infection (Figure 7C), suggesting the entry of PV does not require clathrin-mediated endocytosis. Consistent with this conclusion, imaging of PV in live cells expressing fluorescent clathrin fusion proteins [39] did not reveal significant colocalization of PV with clathrin-coated pits prior to PV entry (unpublished data).

A number of viral entry pathways, including classical caveolin-mediated endocytosis, were reported to be dependent on cholesterol-rich domains or rafts [40,41]. Sensitivity to drugs that deplete cholesterol either by removing it from membranes (e.g., methyl-β-cyclodextrin [MβCD]) or by inhibiting cholesterol synthesis (nystatin and progesterone), or drugs that bind cholesterol and interfere with raft formation (filipin) have therefore often been used as surrogate markers for caveolin-dependent endocytic pathways. Among these drugs, filipin is arguably less prone to downstream effects from bulk depletion of cholesterol. We thus tested the involvement of caveolin-mediated endocytosis using filipin. Our NRIC assay showed that filipin has no effect on PV infection at a concentration of 2 or 20 μg/ml (Figure 7C), concentrations that efficiently block the entry of fluorescently labeled cholera toxin [39,42]. These results suggest that PV enter HeLa cells through a caveolin-independent pathway. We have also tested the effects of MβCD (Figure 7C), nystatin, and progesterone (unpublished data). All of these drugs substantially reduced PV infectivity in both illuminated samples and the nonilluminated controls, suggesting irreversible adverse effect by these drugs on the cells, unrelated to viral entry. This finding is consistent with a previous report by Liu et al [43], and explains why earlier studies using cholesterol-depleting MβCD show inhibited PV infection [41].

Combining clathrin siRNA and filipin to simultaneously inhibit clathrin-mediated endocytosis and caveolin-mediated endocytosis also had no significant effect on PV entry, suggesting that PV enter HeLa cells via a clathrin- and caveolin-independent pathway. Recently, flotillin-1 was identified as a marker protein for a subset of clathrin- and caveolin-independent pathways [42,44]. Knocking down flotillin-1 with a specific siRNA (Figure 7A) showed no effect on the PV infection, but the same knockdown procedure inhibited several cationic ligands that we have shown to enter cells through a clathrin- and caveolin-independent pathway [42], suggesting that PV entry also does not require flotillin-1 (Figure 7C).

Although macropinocytosis has not previously been implicated as a significant pathway in PV entry [45], we also tested the effects of amiloride, a drug that interferes with macropinocytosis by blocking sodium channels. At concentrations (200 μM) that block the uptake of fluorescently labeled dextran though macropinocytosis, amiloride did not affect the ability of PV to infect cells (Figure 7C).

**Figure 8:**
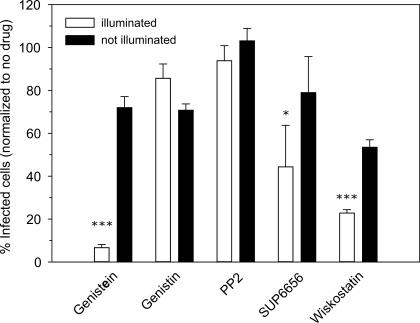
Endocytosis of PV Is Tyrosine Kinase Dependent Effect of various tyrosine kinase inhibitors on PV entry measured by using the NRIC assay. A single asterisk (*) indicates *p* < 0.05; triple asterisks (***) indicate *p* < 0.001.

Tyrosine kinases are often involved in cortical actin remodeling, endocytosis, intracellular trafficking, and various steps of viral infection [46–50]. We therefore tested the effect of tyrosine kinase inhibitors on polio entry by the NRIC assay. A strong inhibitory effect on PV infection was observed with genistein, a well-known tyrosine kinase inhibitor with broad specificity, but not with genistin, an inactive analog often used to screen out nonspecific effects (Figure 8). However, tests with inhibitors that more narrowly impact subfamilies of tyrosine kinase, including PP2 (an Src family inhibitor with high affinity for p56 and Hck), SU6656 (an inhibitor of src family kinases with high affinity for Src), and wiskostatin (an inhibitor of the NWASP, Arf2/3 pathway), exhibited at most a moderate effect on PV infection. Thus, the specific tyrosine kinase(s) involved remain to be identified.

## Discussion

The extracellular forms of viruses face formidable challenges. The virion itself must be sufficiently stable to protect the viral genome during the passage from host to host and cell to cell, and yet, upon reaching the target cell and encountering the appropriate trigger, the virion must initiate programmed steps that result in the release of the viral genome into the appropriate compartment of the cell. For nonenveloped viruses, the conceptually simple mechanism of membrane fusion is not an option. Instead, they must provide the machinery required to translocate either a nucleoprotein complex or, minimally, the viral genome across a cell membrane. Although some of the machinery required to deliver the viral genome must be supplied by the virus itself, it is not surprising, given the limited coding capacity of viral genomes, that most viruses hijack cellular pathways in order to initiate infection. The characterization of the cell entry pathways for nonenveloped viruses has been challenging due to the difficulties associated with direct visualization of the viral gene-delivery process, making it hard to determine exactly when, where, and how the viral genome is released. The situation is further complicated by a number of factors, including the high particle-to-pfu ratio of most animal viruses, which makes it impossible for visualization methods and most biochemical methods to distinguish productive and nonproductive pathways. As a result, the entry pathways of most nonenveloped viruses remain poorly understood. This is true even for viruses like PV, which have been the subject of decades of research, and have long been thought of as models for understanding viral–host interactions.

To overcome the above problems, we developed assays that focus more directly on productive pathways. Toward this end, we established a protocol for labeling viral RNA by growing virus particles in the presence of cell-permeable nucleic acid–binding dyes. The dye labels did not inhibit viral infectivity but provided sufficiently bright signal for single viral RNA imaging in live cells. The simultaneous labeling of viral genome and capsid allowed us to follow individual viral capsids together with their genomic RNA so as to precisely determine when and where the RNA was released from the capsid (Figure 1). To complement this imaging approach, we also refined a NRIC assay to identify inhibitors that perturb early infection steps leading to RNA release (Figure 4). We coupled these two assays to address the PV entry mechanism and to systematically screen the effect of small-molecule inhibitors on PV infection. The complete correspondence of the RNA release kinetics and effects of inhibitors in the two assays provide compelling evidence that the pathways elucidated in the visualization assay are biologically relevant.

Productive release of the viral genome has been previously suggested as a major factor limiting the efficiency of infection [45], but the model has not been rigorously proved or disproved due to the lack of suitable technology. Remarkably, we have observed rapid and highly efficient RNA release (Figures 1 and S2). The close correspondence of the kinetics of RNA release and the effects of inhibitors in the visualization and infectivity assays (Figures 1, 5, and 6) suggest that the observed RNA release is on the pathway leading to infection. This implies that the high particle-to-pfu ratio of PV results from steps after genome release, possibly including degradation of viral RNA by cellular RNases, inefficient initiation of the first round(s) of translation of viral RNA (which is uncapped and requires IRES-dependent initiation) [4], or inefficient assembly of the input RNA into replication complexes.

We have shown that PV must enter the cell first before the RNA release can take place. The requirement of internalization is not only suggested by the observation that the virus particles remaining on the cell surface did not release their RNA (Figure 3), but also supported by the observed dependence of RNA release and subsequent infection on temperature, energy, actin cytoskeleton, and tyrosine kinases (Figures 5–8). It is formally possible that complete vesiculation is not required and that RNA release takes place in a deep invagination inaccessible to pH change in the extracellular medium. However, we do not favor this latter model as this requires a sufficiently tight restriction of the invagination neck to block passage of such miniscule ions as hydrated protons and hydroxyl ions.

Remarkably, RNA release was found to occur almost immediately following internalization of the virus particles extremely close to the cell surface, in vesicles that were located within 100–200 nm of the plasma membrane (Figure 2C). Whereas the site of RNA release was more precisely determined for the adhering surface of the cell by TIRF, we showed, using confocal microscopy, that RNA release took place close to either the top (apical) or the bottom (attached) surface of cells with similar kinetics (Figure 2A). Inhibitors that disrupt actin cytoskeleton, deplete ATP, or inhibit tyrosine kinases totally block infection from both surfaces (Figures 5–8). Thus, the entry pathways from the two surfaces of nonpolarized cells are most likely similar, if not identical.

The demonstration that genome release takes place from vesicles very near the cell surface is consistent with previous observations that endocytic acidification and intact microtubules are not required for PV infection [32,33]. Indeed, we have confirmed that neither raising endocytic pH nor disrupting microtubules inhibits either RNA release or infection (Figure 6). Interestingly, shortly after RNA release, the empty capsids were observed to move rapidly along microtubules. However, this transport behavior is unlikely to be relevant to PV infection. Our results challenge previous thoughts that PV RNA release may occur at the plasma membrane or near the nucleus [45]. They also raise the question of what triggers RNA release from the vesicles near the plasma membrane. One potential candidate arises from the curvature of the vesicles enveloping the viral particles, which may allow additional copies of the N-terminus of VP1 to be inserted into the vesicle membrane or allow more intimate association of the virus particles with the membrane, generating mechanical forces that trigger RNA release. It is also possible that internalization results in the recruitment of cellular factors that facilitate RNA release and translocation across the vesicle membrane. Using well-characterized endocytic inhibitors, we demonstrated that the PV internalization into HeLa cells does not require clathrin- or caveolin-mediated endocytosis, but depends on yet unidentified tyrosine kinase(s) (Figures 7 and 8). This is consistent with earlier observations that a dominant negative mutant for dynamin, a small GTPase involved in both clathrin- and caveolin-mediated endocytosis, does not block the entry of PV [19]. Therefore, although PV particles have been observed in clathrin-coated pits in fixed cells, this represents either a nonproductive or a nonessential pathway. The entry behavior of PV appears to be distinct from other picornaviruses: Foot-and-mouth disease virus enters cell via clathrin-mediated endocytosis and takes advantage of the acidic milieu of endosomes to release genome [51], whereas echovirus 1 enters cells using caveolin-mediated endocytosis and apparently uncoats from the caveosome [52], and Coxsackievirus B3 enters polarized epithelial cells via caveolae and apparently releases its viral genome from the endoplasmic reticulum [49]. Diversity suggests that viruses in the family may be rather promiscuous in their choice of pathways, and suggests a possibility that viral entry mechanisms may be for a given virus dependent on the type of cell being infected.

**Figure 9:**
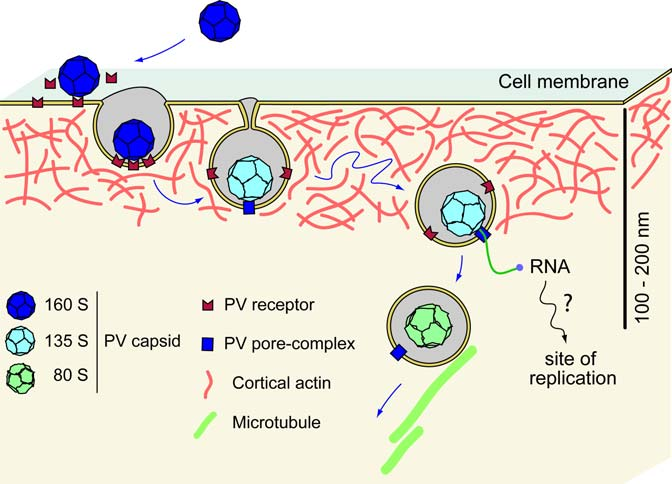
Model of PV Entry After binding to cell-surface receptors, PV (160S) undergoes conformational changes of the capsid to the 135S form. The 135S particles are then internalized by a clathrin- and caveolin-independent, but actin- and tyrosine kinase-dependent, mechanism. The release of the viral genome takes place only after internalization from an endocytotic compartment localized within 100–200 nm of the plasma membrane. Upon release of the RNA genome, the empty capsid (80S) is transported away along microtubules.

Taken together, the results of this study suggest the following model for viral entry and genome release (Figure 9): After binding the cell surface, the virus is internalized through a clathrin-, caveolin-, and flotillin-independent, but actin- and tyrosine kinase-dependent, pathway. After internalization (and only after internalization), the virus releases its RNA rapidly from vesicles that are located within 100–200 nm of the plasma membrane without requiring endocytic acidification or microtubule-dependent transport. Our results have settled the long-lasting debate of whether PV directly breaks the plasma membrane barrier or relies on endocytosis to deliver its genome into the cell. These results have also opened interesting questions for this important virus that await further investigation, including what characteristic of these endocytic vesicles near the plasma membrane triggers RNA release; and after release near the cell surface, how is the released RNA transported to replication sites.

## Materials and Methods

### Cells and media.

Adherent HeLa (S3), Hek 293, and SH-SY5Y cells were maintained in a 5% CO_2_ environment at 37 °C in Dulbecco's modified Eagle medium (DMEM; Invitrogen, http://www.invitrogen.com) supplemented with 10% fetal bovine serum, 1 mM nonessential amino acids, 2.5 mM glutamine, and antibiotics (25 U/ml penicillin and 25 μg/ml streptomycin). Suspension cultures of HeLa (S3) cells were maintained in Joklik's minimal essential medium as previously described [54].

### Preparation of labeled PV.

PV serotype 1, Mahoney strain, was grown in suspension cultures of HeLa cells as previously described [53,54]. To introduce the RNA dye into the virion, Syto 82 (Molecular Probes, http://probes.invitrogen.com) was added to the infected cell culture at 2 h p.i. at a final concentration of 25 μM. Other RNA-binding dyes tested (acridine orange, RiboGreen, RNA Select, and Syto: 80, 81, 83, 85, 11, 12, 13, 14, 15, 16, 17, 59, 61, 63, and 64) did not meet the five-criteria list in the main text and thus not were suitable for the single-virus tracking experiments. To prepare NR-labeled virus, cells were infected in the presence of 10 μg/ml NR. Infected cells were harvested by centrifugation at 5.5–6 h p.i. and virus was released by freeze-thawing the cell pellet twice. Cell debris was removed by low-speed centrifugation, and virus was purified with a CsCl density gradient as previously described [53]. For NR labeling, the viruses were infected and purified in the dark. For labeling the capsid with Cy5 or the pH-sensitive CypHer5 (GE Healthcare, http://www.gehealthcare.com/usen/index.html), purified virus was incubated with the amine-reactive dye in a carbonate buffer (pH 9.3) at 22 °C and gently rocked for 1 h. Unbound dye was removed via buffer exchange into 50 mM Hepes buffer (pH 7.4, 145 mM NaCl) using gel filtration columns (Nap5; GE Healthcare). Virus titers were determined using plaque assays on adherent HeLa (S3) cells [54].

### Live-cell fluorescence microscopy.

Cells, cultured in glass-bottom dishes (MatTek, http://www.mattek.com), were washed twice with PBS, and PV was added to cells at 37 °C. After 5 min of incubation, the unbound viruses were removed and replaced by fresh phenol red–free DMEM (Invitrogen) containing 1% glucose, 50 mM Hepes, an oxygen-scavenging system (glucose oxidase, 0.2 mg/ml [Sigma, http://www.sigmaaldrich.com] and catalase, 1 μg/ml [Roche Diagnostics, http://www.roche.com]). The dish was placed on the stage of a temperature-controlled microscope. Unless otherwise noted, experiments were conducted at 37 °C. At each time point during the infection, a series of images was recorded at several focal planes (3–5 in epifluorescence- and 10–20 in confocal microscopy) within the cell in order to determine the number of PV which have released their RNA.

Unless otherwise noted, cells were infected at a low MOI (1 pfu per cell) of PV. For the virus preparations used in the experiments reported here, this corresponds to about 300–400 of virus particles bound per cell, a density that allows individual virus particles to be readily discerned. In the rare cases in which viruses at certain regions of the cell were too dense, that region was excluded from quantitative analysis. A field of view contained five to ten cells (depending on the cell type).

For pH-dependence microscopy assays, neutral medium (phenol red–free DMEM, 1% glucose, oxygen-scavenging system, 50 mM Hepes [Sigma], pH 7.2) was carefully aspirated and quickly replaced by basic medium (phenol red–free DMEM, 1% glucose, oxygen-scavenging system, 50 mM Capso [Sigma], pH 9.5). Immediately after obtaining the data, the cells were washed with fresh neutral medium. One pH shift lasted not more than 30 s.

Two-color fluorescence images were collected through a 1.4 N.A. oil-immersion objective (Olympus, http://www.olympus.co.jp/en/), separated by 650 nm long-pass dichroic mirrors (Chroma, http://www.chroma.com), and imaged onto two separate areas of a charge-coupled device (CCD) camera (CoolSNAP HQ; Roper Scientific, http://roperscientific.com). Capsid signals (Cy5 or CypHer5) were detected using continuous excitation with 633 nm HeNe laser (Melles-Griot, http://www.mellesgriot.com), and their signals were collected through a 665 nm long-pass filter (Chroma). The Syto82 signals were excited with a 532 nm Nd:YAG laser (Crystalaser, http://www.crystalaser.com) strobed at 1 Hz. The strobing reduced the laser illumination time and thus avoided photobleaching of Syto82. It also allowed the separate analysis of Cy5 (viral capsid) signals without the cellular background generated at 532 nm and the crosstalk of Syto82. Syto82 signals were collected through a 585/35 nm bandpass filter (Chroma). All images were acquired at 2 Hz for the Cy5 signal and 1 Hz for the Syto82 signal. Differential interference contrast (DIC) optics (Olympus) was used to obtain cell images before and after the fluorescence imaging. Experiments were conducted at 37°C.

### Image analysis.

Image analysis was carried out using custom-written IDL software as previously described [39] (including a Fourier bandpass filter and convolution with a Gaussian function, the width of which was twice the size of the point spread function of the microscope, both of which reduce background signals and noise). Colocalization of RNA and viral capsid signals was identified by an automated program and confirmed by eye. Objects were scored as colocalized if the distance between the center of the fluorescence image of the RNA and the fluorescence image of the capsid was less than four pixels. To estimate the error in the calculated colocalization fraction due to overlap by chance, randomly distributed spots of each color with the experimentally determined point spread function were analyzed and resulted in a colocalization fraction of smaller than 10% due to random overlap.

### Drugs and silencing RNA.

Working concentrations of the drugs are noted in parentheses. R78206 (10 μg/ml) was a generous gift from Koen Andries (Janssen Pharmaceuticals). Nocodazole (10 μg/ml), brefeldin A (10 μg/ml), bafilomycin A1 (320 nM), Cyto-D (10 μg/ml), genistein (12.5–187.5 μg/ml), genistin (8–120 μg/ml), SU6656 (1–30 mM), chlorpromazine (0.5–20 μg/ml), filipin (2–20 μg/ml), sodium azide (20 mM), 2-deoxyglucose (50 mM), and amiloride (200 μM)were purchased from Sigma. PP2 (1–20 μM) and wiskostatin (5–20 μM) were purchased from EMD Biosciences ( http://www.emdbiosciences.com/home.asp). siRNA for the clathrin heavy chain (CLTC, SMARTpool L-004001-00-0005) and the μ1 subunit of adaptor protein 2 (AP2M1, SMARTpool L-008170-00-0005) were purchased from Dharmacon (http://www.dharmacon.com). Flotillin-1 siRNA and control siRNA were obtained from Santa Cruz Biotechnology (http://www.scbt.com) Cells were transfected at a final concentration of 5 nM at 2 and 1 day preinfection.

### Neutral red infectious center assay.

Monolayers of cells were pretreated with a drug in serum-free media for 1 h. In the dark, cells were infected at an MOI equal to one. Virus adsorption was performed at 12 °C for 30 min in the presence of the drug. Nonbound virus was rinsed off with cold PBS. Infection was initiated with the addition of warm, drug-containing medium and continued at 37 °C. At 30 min p.i., infected monolayers are exposed to white light (transilluminator; VWR, http://www.vwrsp.com) for 10 min at room temperature, while duplicate control monolayers are maintained in the dark. Cells were then trypsinized and counted. Known dilutions of the infected cells in drug-free, complete medium were plated onto new monolayers of cells and allowed to adhere. At 4 h p.i., the medium was replaced with an agar overlay. The number of plaques, each representing an infected cell from the original monolayer, was counted at 36–48 h p.i.. For cells treated with siRNA, drugs were omitted in all steps.

### Western blot analysis.

Western blot analyses were performed as previously described [55]. CLTC was detected using a 1:1,000 dilution of mouse anti-CLTC monoclonal antibody (BD Biosciences http://www.bdbiosciences.com). AP2M1 was detected using a 1:1,000 dilution of mouse anti-AP50 monoclonal antibody (BD Biosciences). α-Tubulin was detected using a 1:2,000 dilution of mouse anti–α-tubulin monoclonal antibody (Invitrogen). Flotillin was detected using a 1:1,000 dilution of a mouse anti-flotillin-1 (H-104) polyclonal antibody (Santa Cruz Biotechnology).

## Supporting Information

Figure S1 Metabolic Labeling of PV RNA with Syto 82 or Neutral Red Does Not Reduce the Infectivity White light illumination of NR-labeled, but not of Cy5 or Syto82, PV leads to photoinactivation and significant reduction of specific infectivity.
(149 KB TIF)

Figure S2 Time Lapse Images of PV RNA Release Live HeLa cell were incubated with dual-labeled PV (RNA with Syto82, green, and capsid with Cy5, red) (MOI = 1) and the RNA release was observed on a single-particle level in the same cell over time. The cell boundaries were traced with blue lines, and the nuclei were marked with “N.”
(A) Wide-field illumination (epi) of the bottom focal plane of the cell.
(B) To confirm that the disappearance of the RNA signal was not due to photobleaching, the experiment was conducted at room temperature (24 °C) at which the structural conversion of the capsid (160S to 135S) was significantly inhibited. Scale bar indicates 10 μm.
(C) RNA release over time observed in the same cell using TIRF imaging geometry.
(4.5 MB TIF)

## Abbreviations

CLTC: clathrin heavy chain
Cyto-D: cytochalasin D
MOI: multiplicity of infection
NR: neutral red
NRIC: neutral red infectious center
pfu: plaque forming unit
p.i.: postinfection
PV: poliovirus
PVR: poliovirus receptor (CD155)
siRNA: small interfering RNA
TIRF: total internal reflection fluorescence
VP: viral capsid protein
