# Supplementary figures and images for: Imaging Poliovirus Entry in Live Cells
Source: PLoS Biol. 2007 Jul 10;5(7):e183. doi: 10.1371/journal.pbio.0050183 (PMC1914398; doi:10.1371/journal.pbio.0050183)

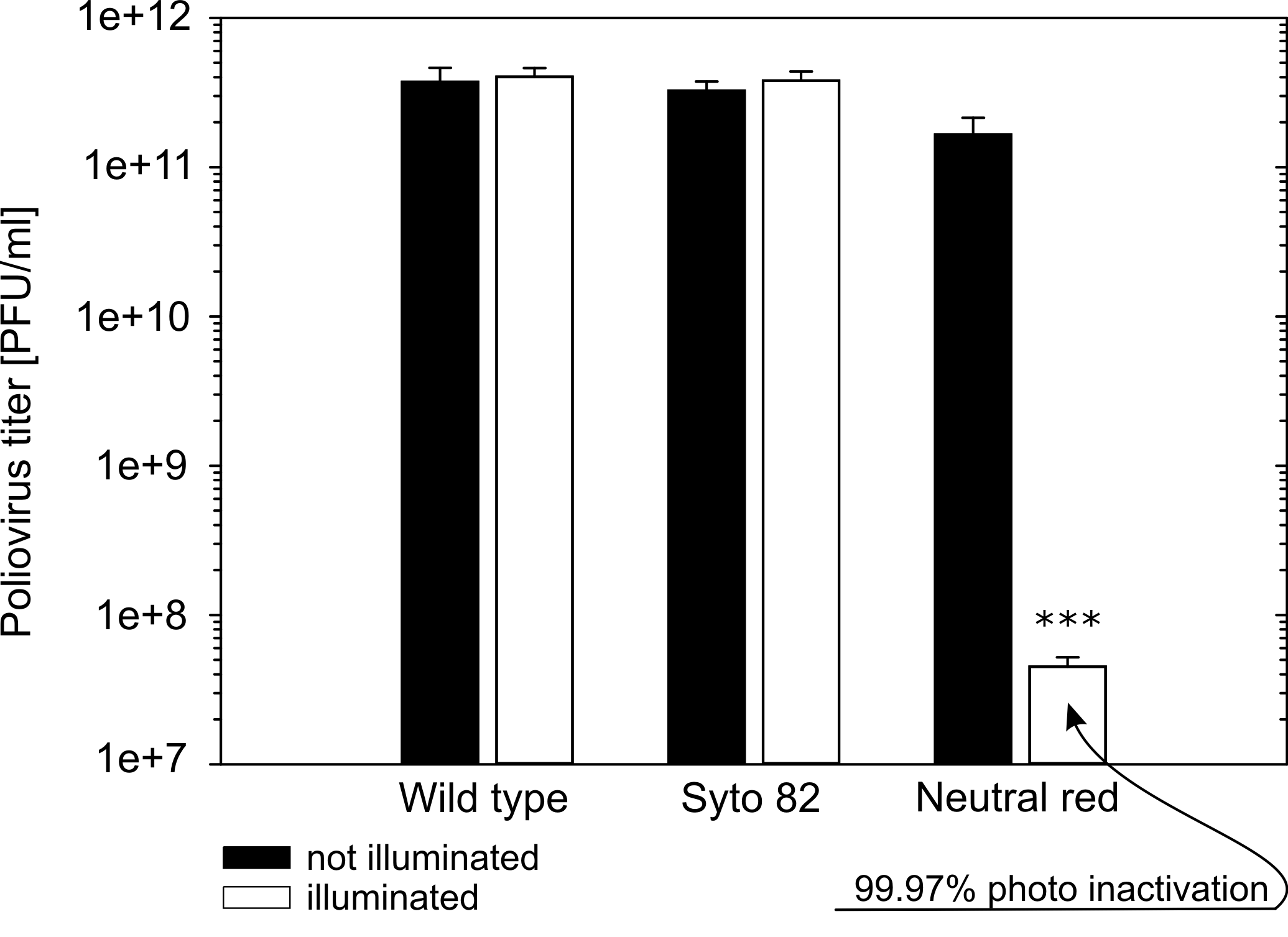

Supplement: Figure S1 — White light illumination of NR-labeled, but not of Cy5 or Syto82, PV leads to photoinactivation and significant reduction of specific infectivity. (149 KB TIF) [file pbio.0050183.sg001.tif]

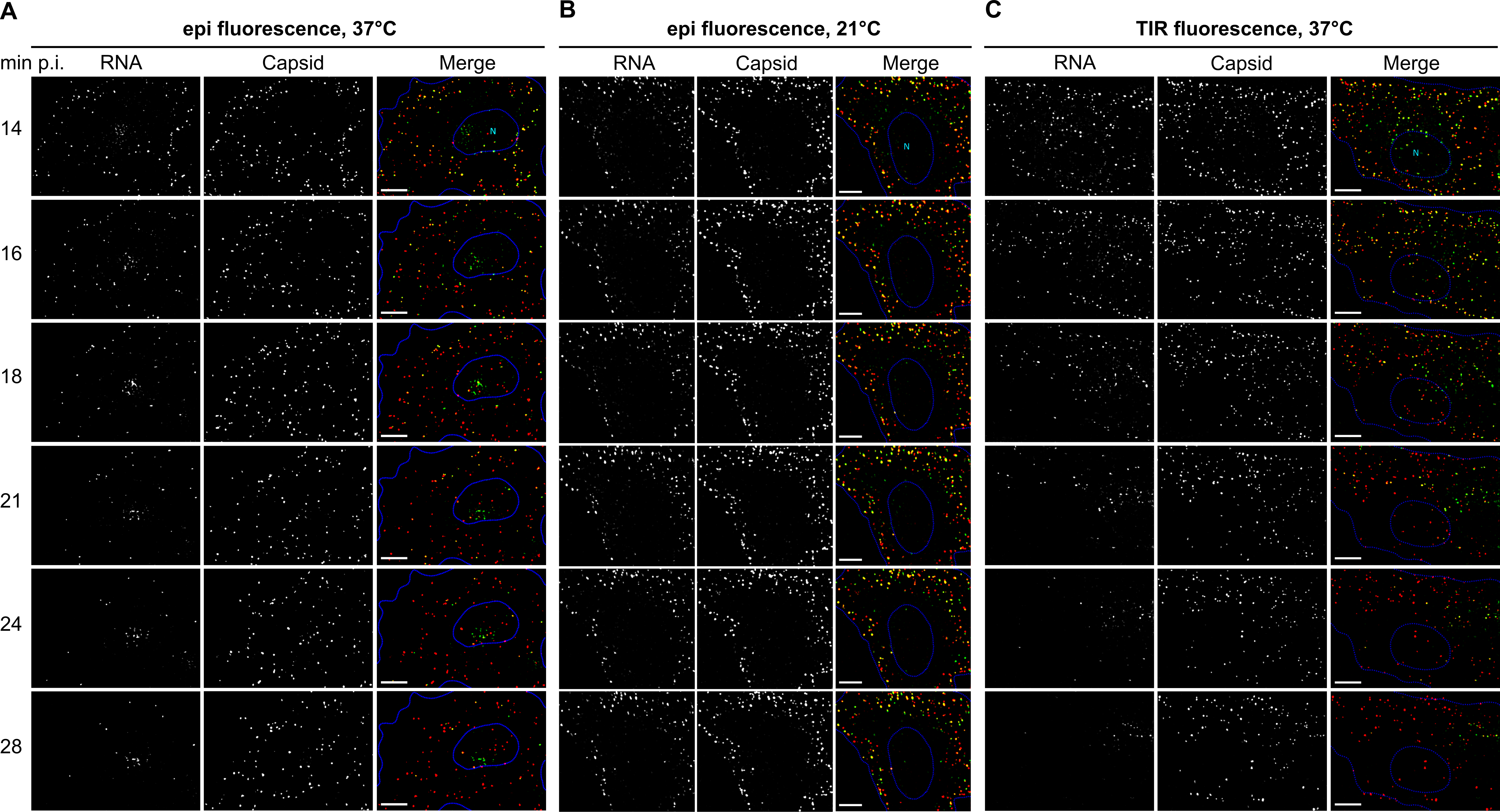

Supplement: Figure S2 — Live HeLa cell were incubated with dual-labeled PV (RNA with Syto82, green, and capsid with Cy5, red) (MOI = 1) and the RNA release was observed on a single-particle level in the same cell over time. The cell boundaries were traced with blue lines, and the nuclei were marked with “N.” (A) Wide-field illumination (epi) of the bottom focal plane of the cell. (B) To confirm that the disappearance of the RNA signal was not due to photobleaching, the experiment was conducted at room temperature (24 °C) at which the structural conversion of the capsid (160S to 135S) was significantly inhibited. Scale bar indicates 10 μm. (C) RNA release over time observed in the same cell using TIRF imaging geometry. (4.5 MB TIF) [file pbio.0050183.sg002.tif]
